# Epidemiological Characteristics and Treatment Outcomes of Drug-Resistant Tuberculosis in Limpopo Province, South Africa (2020–2024)

**DOI:** 10.3390/tropicalmed11040100

**Published:** 2026-04-13

**Authors:** Ivy Rukasha, Kabelo Gabriel Kaapu

**Affiliations:** Division of Medical Microbiology, Department of Pathology, School of Medicine, University of Limpopo, Sovenga 0727, Limpopo, South Africa

**Keywords:** drug-resistant TB, Limpopo, treatment outcomes, rifampicin-resistant TB, HIV, South Africa, 2024

## Abstract

**Background**: Drug-resistant tuberculosis (DR-TB) continues to pose a major challenge in Limpopo Province, a predominantly rural region of South Africa with high prevalence of HIV and mobility of the cross-border population. Despite the scale-up of short all-oral bedaquiline-based regimens, there is limited recent provincial evidence describing DR-TB epidemiological characteristics and treatment outcomes in the post-COVID-19 period. This study aimed to assess resistance patterns, treatment outcomes, and factors associated with unfavorable outcomes among patients with DR-TB in Limpopo Province from 2020 to 2024. **Methods**: A retrospective cohort study was conducted using routinely collected data from the Electronic Drug Resistant Tuberculosis Register (EDRWeb). All laboratory-confirmed DR-TB cases diagnosed between January 2020 and December 2024 were included. Descriptive statistics were used to summarize demographic and clinical characteristics. Multivariable logistic regression was performed to identify predictors of unfavorable outcomes (treatment failure, death, and loss to follow-up). Kaplan–Meier survival analysis was used to estimate survival probability following treatment initiation. **Results**: A total of 1240 DR-TB cases were recorded, of which 1165 (94%) had documented treatment outcomes. Rifampicin-resistant TB (RR-TB) predominated throughout the study period, accounting for 76% (951/1240) of cases and remaining stable over time. Treatment success improved from 173/260 (67%) in 2020 to 130/166 (78%) in 2024, while loss to follow-up declined from 34/260 (13%) to 4/166 (2%). Kaplan–Meier survival analysis showed that mortality occurred predominantly during the early phase of treatment. Patients receiving bedaquiline-containing regimens demonstrated significantly higher survival probability compared with those not receiving bedaquiline (log-rank *p* = 0.024; HR 0.58, 95% CI: 0.35–0.94). In multivariable analysis, HIV infection was independently associated with unfavorable outcomes (aOR 1.36; 95% CI: 1.04–1.77; *p* = 0.025), while increasing age showed a modest association with poorer outcomes. **Conclusions**: Treatment outcomes for DR-TB improved over the study period, accompanied by declining loss to follow-up and improved survival. The survival advantage observed among patients receiving bedaquiline-containing regimens supports continued prioritization of bedaquiline-based treatment strategies in DR-TB management. Strengthening access to these regimens, alongside integrated HIV care, may further improve treatment outcomes in Limpopo Province and similar high-burden settings in South Africa.

## 1. Introduction

Tuberculosis (TB) remains the leading infectious cause of death in the world. The World Health Organization (WHO) Global Tuberculosis Report 2024 estimates that 10.8 million people developed TB in 2023 and 1.25 million died from the disease [1]. Drug-resistant tuberculosis (DR-TB) remains a growing public health concern in sub-Saharan Africa. The World Health Organization (WHO) estimates that approximately 62,000 new cases of multidrug-resistant tuberculosis (MDR-TB) occurred in the African region in 2022. Although the proportion of rifampicin resistant RR-TB/MDR-TB among newly diagnosed TB cases in the WHO African region is estimated at around 2%, the burden is substantially higher among previously treated patients, highlighting the role of treatment failure and inadequate adherence in driving resistance. Southern Africa, including South Africa, accounts for some of the highest numbers of DR-TB cases on the continent [2,3].

South Africa remains one of the countries with the highest burden of DR-TB. In 2023, an estimated 270,000 people developed TB nationally, including approximately 13,000 (5%) with DR-TB. Coinfection of TB/HIV within the country accounts for more than half (over 54%) of all TB cases [1,4]. Although substantial national progress has been made through the introduction of GeneXpert MTB/RIF testing, the implementation of all oral bedaquiline (BDQ)-containing regimens, and the decentralization of DR-TB services, major provincial disparities persist [5,6]. Limpopo Province, situated in the northern region of South Africa, represents a particularly vulnerable setting due to its predominantly rural geography, high HIV prevalence, limited diagnostic infrastructure and significant population mobility. Limpopo Province also faces a high burden of HIV infection, with an estimated HIV prevalence of approximately 17–19% among adults, which further complicates TB control efforts and increases the risk of poor treatment outcomes [7]. Notably, Limpopo is a border province that shares extensive boundaries with Zimbabwe, Mozambique, and Botswana, which further intensifies challenges related to migration, health surveillance, and continuity of care [8,9]. These factors collectively increase the risk of delayed diagnosis and sustained transmission of resistant strains of *Mycobacterium tuberculosis.*

Previous reports of DR-TB in Limpopo have highlighted several important trends: persistently high proportions of RR-TB and MDR-TB, with only modest improvements in treatment success, and disproportionately unfavorable outcomes among HIV co-infected patients and those with advanced resistance [6,8,9,10,11,12]. More recent analyses of BDQ-containing regimens (2016–2019) in the province further demonstrated improved survival and faster culture conversion among patients receiving BDQ, reaffirming its critical role in optimizing outcomes in rural settings [6]. Other studies have revealed concerning levels of fluoroquinolone and BDQ resistance, along with evidence of local and interdistrict transmission clusters within the province, underscoring the growing threat of both acquired and transmitted drug resistance in the region [9].

Although Seloma et al. (2023), Rukasha et al. (2025), and Kaapu et al. (2025) have provided crucial provincial insights into TB patterns, there remains a gap in recent epidemiological data describing epidemiological characteristics and treatment outcomes in Limpopo in the era of widespread implementation of short all-oral regimens [6,8,10]. Understanding the current patterns of disease, the profile of affected patients, and the factors driving treatment outcomes is essential for informing provincial TB control strategies, particularly in settings with similar health system constraints, high HIV burden, and cross-border mobility dynamics.

The objective of this study was to describe the epidemiological patterns of drug-resistant tuberculosis in Limpopo Province between 2020 and 2024, assess treatment outcomes and factors associated with unfavorable outcomes, and evaluate overall survival following treatment initiation using Kaplan–Meier survival analysis. By providing up-to-date evidence from a rural province with high mobility and limited resources, the findings provide information relevant to South Africa and other regions facing persistent transmission of DRTB, high burden of HIV, fragile health systems, and challenges in the implementation of short all-oral regimens.

## 2. Materials and Methods

### 2.1. Study Approvals

Ethical approval for this study was obtained from the Turfloop Research Ethics Committee (TREC) of the University of Limpopo (Reference: TREC/108/2024: PG) and the Limpopo Department of Health (Reference: LP_2024-12-011). The provincial health authorities granted permission to access and use routine programmatic data. All data were anonymized prior to analysis, and confidentiality and privacy of patient information was strictly maintained throughout the study.

### 2.2. Study Design

This study followed a retrospective cohort design that used routinely collected data from the Electronic Drug-Resistant Tuberculosis Register (EDRWeb) for the period January 2020 to December 2024. All laboratory-confirmed cases of DR-TB recorded in Limpopo Province during this period were included. The study aimed to assess epidemiological characteristics in DR-TB notifications and to examine associations between demographic and clinical characteristics and treatment outcomes. Data extracted from EDRWeb included patient age, sex, HIV status, type of tuberculosis, resistance profile, treatment history, treatment regimen, and final treatment outcome (categorized as cure, treatment completion, death, treatment failure, or loss to follow-up). Descriptive statistics were used to summarize the data, while chi-square tests and binary logistic regression analyses were applied to explore factors independently associated with unfavorable treatment outcomes. In addition to treatment outcome analysis, a survival analysis was conducted to evaluate overall survival following treatment initiation. Survival time was defined as the number of days from DR-TB treatment initiation to death. Patients who were cured, completed treatment, or remained on treatment at the end of follow-up were treated as censored observations. Kaplan–Meier methods were used to estimate survival probabilities over time and to visualize mortality patterns during the treatment period.

### 2.3. Study Site

**Figure 1:**
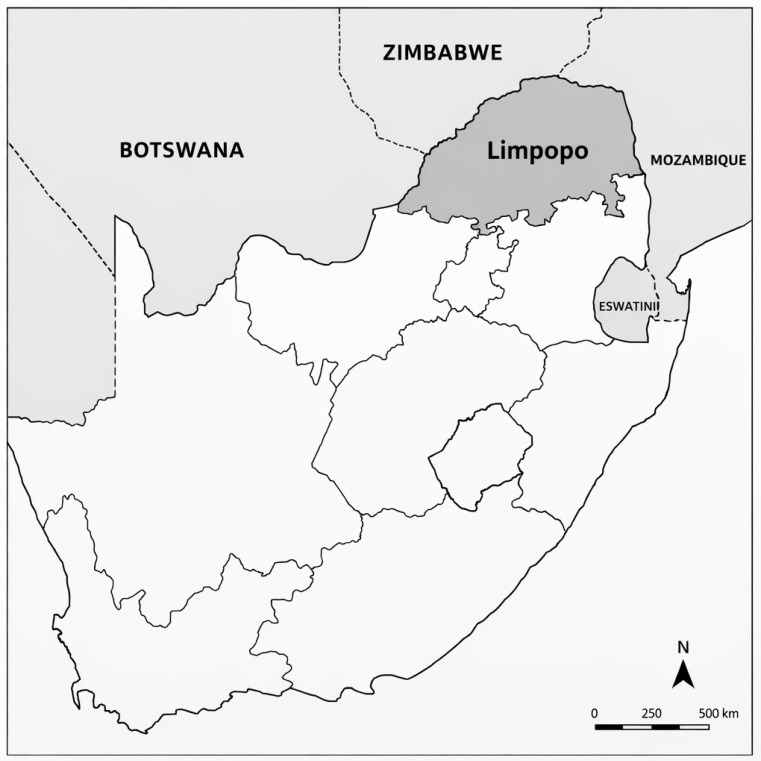
A schematic map of South Africa showing the location of Limpopo province. Source: Authors.

The study was carried out in Limpopo Province, located in the northern region of South Africa (Figure 1). Limpopo is the fifth largest province in the country, with an estimated population of 6,165,877 in 2024, representing approximately 9.9% of South Africa’s total population of 62,197,933, according to the District Health Barometer [13]. Limpopo Province is located in northern South Africa and shares international borders with Zimbabwe, Mozambique, and Botswana, which contributes to significant cross-border population movement and presents additional challenges for tuberculosis surveillance and continuity of care. Limpopo Province is predominantly rural, with over 87% of its population residing outside urban centers, and continues to face persistent challenges related to healthcare access and infrastructure [10]. Limpopo has been one of the provinces most affected by tuberculosis in South Africa. The region also has a high HIV prevalence, 70% of TB patients in Limpopo are co-infected with HIV, further complicating TB control and treatment. These epidemiological and contextual factors, particularly the overlap of HIV and DR-TB, are known to impact treatment adherence and clinical outcomes in the province.

### 2.4. Data Collection

Data were extracted from the Electronic Drug-Resistant Tuberculosis Register (EDRWeb), the national surveillance system used to monitor DR-TB cases in South Africa. To ensure accuracy, extracted records were cross-checked against the original database fields and verified for completeness and consistency before analysis. The dataset included all laboratory-confirmed DR-TB patients recorded in Limpopo Province between January 2020 and December 2024. The database was extracted on 13 June 2025, and demographic, clinical, treatment, and outcome variables were retrieved for analysis. For each patient, information was extracted on age, sex, HIV status, type of TB (pulmonary or extrapulmonary), resistance profile, treatment history, treatment regimen, and treatment outcome. Outcomes were categorized as cured, treatment completed, treatment failed, died, or lost to follow-up, according to WHO definitions [14].

Extracted data were anonymized and exported into Microsoft Excel before being imported into the statistical software environment for analysis. Data cleaning procedures included checking for duplicate entries, inconsistencies in variable coding, and out-of-range values. Categorical variables were standardized, and derived variables were generated where necessary for analysis. Some records in the database lacked final treatment outcome information at the time of data extraction. This occurred primarily when patients had not yet completed treatment or had been transferred to another treatment facility (“moved out”), where the final outcome was not recorded in the provincial database. Records with missing outcome information were excluded from the outcome analysis, but retained for descriptive and trend analyses to ensure a complete representation of the DR-TB patterns in the province.

### 2.5. Definitions and Treatment Outcomes

RR-TB refers to TB resistant to rifampicin only. MDR-TB refers to TB that is resistant to both rifampicin and isoniazid. Pre-extensively drug resistant TB (Pre-XDR-TB) refers to MDR-TB that is further resistant to fluoroquinolones. Extensively drug resistant TB (XDR-TB) fulfils the definition of Pre-XDR-TB and is further resistant to at least one Group A antituberculosis drug [15]. Treatment outcomes were classified according to the latest WHO definitions and grouped as favorable or unfavorable [14].

The favorable results included patients who were cured or had completed treatment. Cured: a patient with bacteriologically confirmed TB at the beginning of treatment who completed the treatment as recommended by national guidelines, showing documented bacteriological conversion and no evidence of treatment failure. Treatment completed: a patient who completed therapy according to national policy but without sufficient bacteriological evidence to meet the criteria for cure or failure.

Unfavorable outcomes included failure of treatment, death, and loss of follow-up (LTFU). Treatment failure: a patient whose regimen was permanently discontinued or changed to a new treatment strategy due to a poor clinical or bacteriological response. Death: any patient who died before starting treatment or during the course of treatment, regardless of the cause. Loss to follow-up: a patient who did not initiate treatment or whose treatment was interrupted for two or more consecutive months.

### 2.6. Inclusion and Exclusion Criteria

All laboratory confirmed DR-TB cases recorded in the EDRWeb for Limpopo Province between January 2020 and December 2024 were considered for inclusion. Cases were eligible regardless of age, sex, or HIV status, provided they had confirmed resistance to at least one antituberculosis drug.

Inclusion criteria comprised all patients with laboratory confirmed DR-TB (pulmonary or extrapulmonary) who had complete demographic, clinical, and treatment outcome data captured in the EDRWeb system. Exclusion criteria were applied to patients with missing treatment outcome information, incomplete data entries, or unknown DR-TB classification. However, patients who were still on treatment at the time of data extraction were retained in the data set to ensure comprehensive representation of ongoing cases. Patients recorded as “Transfer In” or “Moved In” in the EDRWeb database represented individuals who initiated DR-TB treatment at another facility and were subsequently transferred into a treatment unit within Limpopo Province. These patients were included in the study cohort if their treatment start date, treatment course, and outcome information were available in the database. Records lacking sufficient treatment or outcome information were excluded from outcome regression analyses to avoid misclassification of treatment outcomes.

Of the 1240 DR-TB cases identified, 1165 (94.0%) had complete treatment outcome data and were included in the treatment outcome and regression analyses. A total of 75 cases (6.0%) were excluded from regression analyses due to missing or incomplete outcome data, primarily resulting from patients who were transferred out or still on treatment at the time of data extraction.

For the survival analysis, these patients were retained and treated as right-censored observations at their last recorded follow-up time.

### 2.7. Data Analysis

Data were cleaned and coded using Microsoft Excel and subsequently exported to IBM SPSS Statistics version 30.0 for analysis and cross-validated using R scripts to ensure reproducibility. Continuous variables were assessed for normality using the Shapiro–Wilk test prior to analysis. Normally distributed variables were summarized using means and standard deviations (SD), while non-normally distributed variables were summarized using medians and interquartile ranges (IQR). Categorical variables were summarized using frequencies and percentages, and 95% confidence intervals (95% CI) were calculated for key proportions to provide estimates of the precision of observed frequencies. Associations between categorical variables were evaluated using the Chi-square test; when the assumption of expected cell counts <5 was violated, Fisher’s exact test was applied. A *p*-value < 0.05 was considered statistically significant and effect sizes (Cramer’s V) were reported to quantify associations. For the treatment outcome analysis, patients with missing or transferred outcomes were excluded from the logistic regression models to ensure accurate classification of final outcomes. For the survival analysis, however, these patients were retained in the cohort and treated as right-censored observations at their last recorded follow-up time (days on treatment). Death was defined as the event of interest, while patients who were cured, completed treatment, transferred out, or remained on treatment at the time of database closure were treated as censored observations. Survival analysis was performed using the Kaplan–Meier method to estimate overall survival among DR-TB patients. Time-to-event was calculated as days from treatment initiation to death. Patients who were cured, completed treatment, or remained on treatment were censored at their last follow-up. Kaplan–Meier curves were generated to visualize survival probabilities over time and to assess mortality patterns during treatment. Time-to-event was defined as the number of days from treatment initiation to death. Patients who were cured, completed treatment, transferred out, or remained on treatment at the time of database closure were right-censored at their last recorded follow-up date. Survival curves stratified by bedaquiline use were compared using the log-rank test. A Cox proportional hazards regression model was used to estimate the hazard ratio (HR) and 95% confidence intervals for the association between bedaquiline use and mortality.

To identify factors independently associated with unfavorable treatment outcomes, multivariate logistic regression analysis was performed. Both crude and adjusted odds ratios (OR) with 95% confidence intervals (CI) were reported. The adjusted model included key covariates such as age group, sex, HIV status, resistance profile, and treatment category. All analyses were two-tailed, and the statistical significance was established at *p* < 0.05. Reporting was done according to the STROBE guidelines to ensure transparency and reproducibility. Missing data were minimal across variables. Cases with missing values (e.g., unknown HIV status) were excluded from regression analyses using a complete-case approach, as the proportion of missing observations was very small and unlikely to influence the overall estimates.

## 3. Results

### 3.1. Demographic Characteristics

A total of 1240 cases of DR-TB were recorded; 1165 (94%; 95% CI: 92.5–95.2) had documented treatment outcomes. The median age of the patients was 39 years (IQR: 29–49), with ages ranging from <1 year to 88 years. The majority of cases were male (767/1240; 61.9%, 95% CI: 59.2–64.5), while 473 (38.1%) were female. The age group 35 to 44 represented 32.1% (399/1240) of the patients (Table 1).

### 3.2. DR-TB Distribution

**Figure 2:**
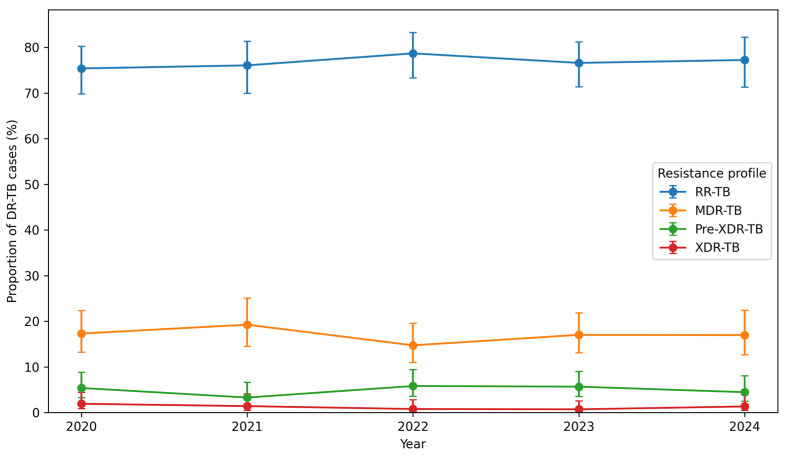
Distribution of RR-TB, MDR-TB, pre-XDR-TB, and XDR-TB in Limpopo Province from 2020–2024. Points represent annual proportions of cases, and vertical bars indicate 95% confidence intervals for each estimate.

Figure 2 shows the annual distribution of DR-TB types between 2020 and 2024. RR-TB remained the predominant form of DR-TB in Limpopo Province, accounting for 75.4% of cases in 2020 (196/260; 95% CI: 69.8–80.2), 76.1% in 2021 (162/213; 95% CI: 69.9–81.3), 78.8% in 2022 (203/258; 95% CI: 73.3–83.2), 76.6% in 2023 (216/282; 95% CI: 71.3–81.2), and 76.7% in 2024 (174/227; 95% CI: 70.7–81.7). MDR-TB accounted for between 14.7% and 19.2% of cases during the study period, while pre-XDR-TB ranged from 3.3% to 8.0%. XDR-TB remained rare, with proportions ranging from 0.7% to 1.9% across the study years. Overall, the five-year distribution shows a predominance of RR-TB with relatively small year-to-year changes, while the proportions of MDR-TB and Pre-XDR-TB fluctuated within narrow ranges. Importantly, XDR-TB remained consistently low, indicating limited transmission or the emergence of highly resistant strains during the study period.

### 3.3. DR-TB Treatment Outcomes

**Figure 3:**
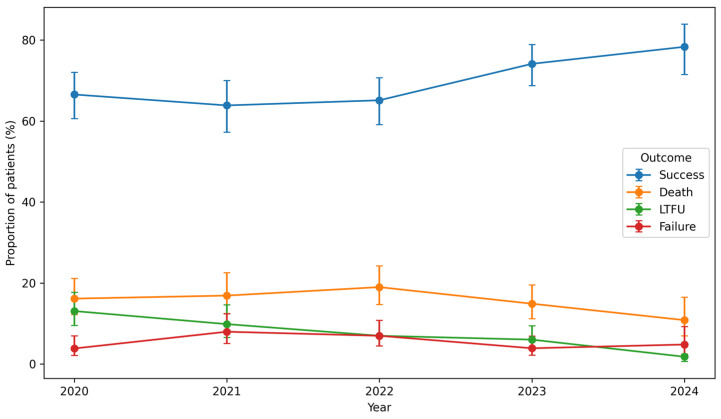
Distribution of DR-TB treatment outcomes in Limpopo Province (2020–2024). Points represent annual proportions of treatment success, death, loss to follow-up, and treatment failure; error bars indicate 95% confidence intervals.

Figure 3 presents the treatment outcomes between 2020 and 2024, demonstrating an overall improvement in treatment performance. Treatment success increased from 173/260 (67%; 95% CI: 61–73) in 2020 to 130/166 (78%; 95% CI: 71–84) in 2024, and this improvement was statistically significant (*p* = 0.006).

Loss to follow-up decreased markedly from 34/260 (13%) in 2020 to 3/166 (2%) in 2024, representing a highly significant reduction (*p* < 0.0001).

Although mortality declined from 42/260 (16%; 95% CI: 12–21) in 2020 to 18/166 (11%; 95% CI: 7–16) in 2024, this change was not statistically significant (*p* = 0.63).

Treatment failure remained relatively low throughout the study period, increasing from 11/260 (4.2%) in 2020 to 15/166 (9.0%) in 2024.

Overall, the proportion of patients achieving successful DR-TB treatment outcomes improved significantly during the study period, accompanied by a substantial reduction in loss to follow-up, while changes in mortality were not statistically significant.

### 3.4. Treatment Success Rates per Resistance Category

**Figure 4:**
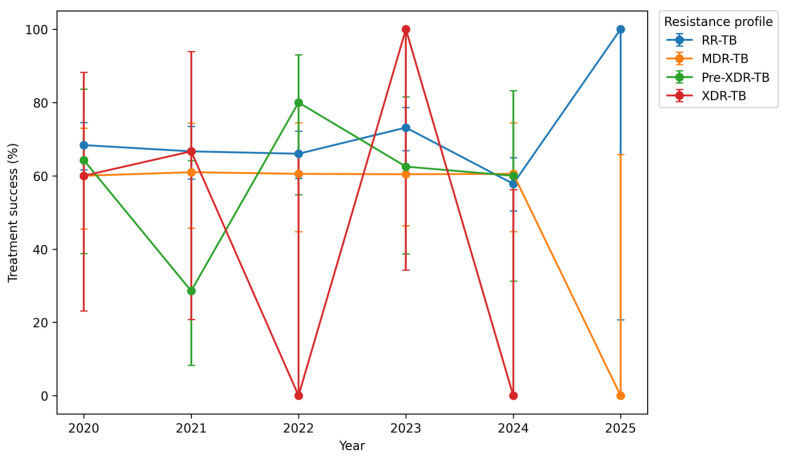
Treatment success rates among DR-TB patients by resistance profile in Limpopo Province (2020–2024). Points represent yearly proportions; error bars indicate 95% confidence intervals.

Figure 4 illustrates treatment success rates by resistance category between 2020 and 2024. Among patients with RR-TB, treatment success remained relatively stable during the early study period, from 176/260 (67.7%; 95% CI: 61.7–73.2) in 2020 to 169/258 (65.5%; 95% CI: 59.5–71.1) in 2022, reaching 130/166 (78.3%; 95% CI: 71.4–84.0) in 2024.

Treatment success among MDR-TB patients ranged from 25/42 (59.5%; 95% CI: 44.8–72.7) in 2020 to 23/29 (79.3%; 95% CI: 60.3–91.1) in 2024.

Success rates among patients with pre-XDR-TB fluctuated across the study period, from 64.3% (9/14; 95% CI: 38.8–83.7) in 2020 to 28.6% (2/7; 95% CI: 8.2–64.1) in 2021, and 80.0% (12/15; 95% CI: 54.8–93.0) in 2022, remaining above 70% during 2023–2024. However, the number of patients in the pre-XDR-TB and XDR-TB categories was small, resulting in wide confidence intervals and increased statistical uncertainty; therefore, these estimates should be interpreted with caution.

Overall, treatment success rates differed numerically across resistance categories, with RR-TB generally showing higher success proportions compared to MDR-TB, pre-XDR-TB, and XDR-TB. However, these differences were not statistically significant (Fisher’s exact test, *p* = 0.108), indicating that the observed variations may reflect random variation rather than true differences between groups. No statistically significant temporal trends were observed across resistance categories.

### 3.5. Associations Between Demographic, Clinical, and Treatment Outcome Variables

**Figure 5:**
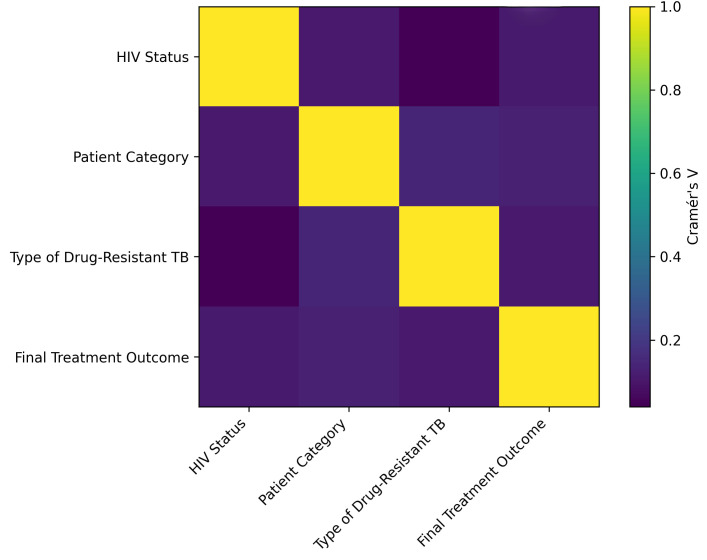
Association matrix showing Cramér’s V coefficients between categorical variables in the DR-TB cohort. Values indicate the strength of association derived from Chi-square contingency analysis.

Associations between key categorical variables were explored using Cramér’s V coefficients derived from Chi-square contingency analysis (Figure 5). Overall, the strength of association between variables was weak to negligible (Cramér’s V < 0.20). A relatively stronger association was observed between patient category and previous treatment history, reflecting their programmatic relationship in the classification of DR-TB cases. Other variables, including HIV status, drug-resistance category, and final treatment outcome, demonstrated weak associations, indicating limited dependence between these factors in the study cohort.

### 3.6. Overall Survival of Patients

**Figure 6:**
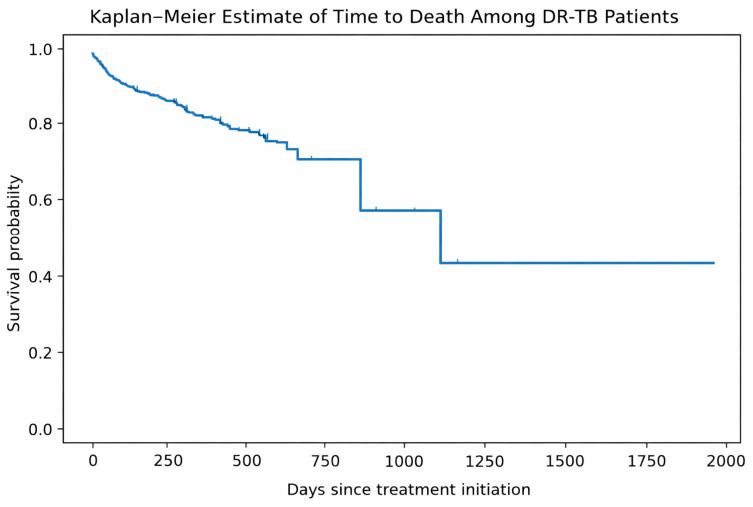
Kaplan–Meier estimate of time to death among drug-resistant tuberculosis (DR-TB) patients following treatment initiation. Death was defined as the event of interest, while patients who were cured, completed treatment, lost to follow-up, transferred out, or remained on treatment were treated as right-censored observations.

Kaplan–Meier analysis was performed to estimate time to death among DR-TB patients following treatment initiation (Figure 6), with death defined as the event of interest and non-fatal outcomes treated as right-censored observations. The survival curve shows an initial decline within the first 60–90 days of treatment, indicating that mortality was concentrated in the early phase after treatment initiation. Thereafter, the rate of decline became more gradual, with relatively few deaths occurring later in the treatment course.

Because a large proportion of patients experienced treatment success or remained on treatment, the majority of observations were censored. Therefore, the Kaplan–Meier curve reflects the timing of mortality events rather than the overall probability of death. The median survival time was not reached during the follow-up period, indicating that more than half of the patients remained alive throughout observation.

### 3.7. Survival Stratified by Bedaquiline Use

**Figure 7:**
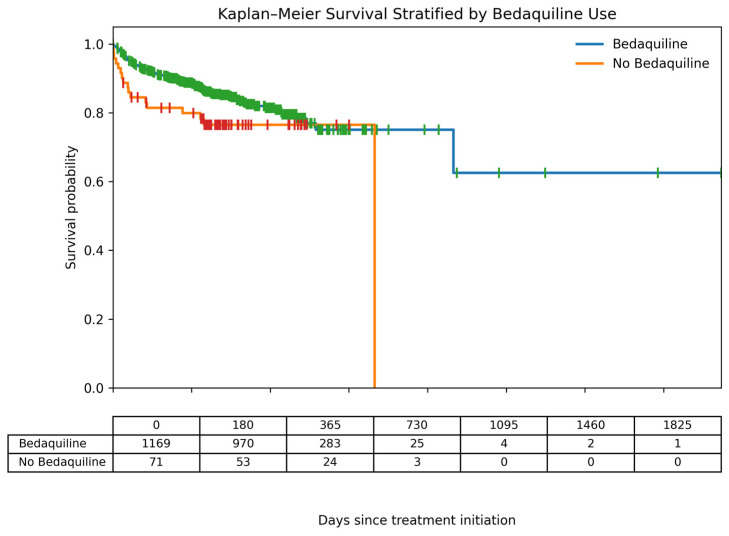
Kaplan–Meier survival curves stratified by bedaquiline use among DR-TB patients. Death was treated as the event of interest, while patients who were cured, completed treatment, lost to follow-up, transferred out, or remained alive at the end of follow-up were treated as right-censored observations. Differences between survival curves were evaluated using the log-rank test (*p* = 0.024).

Kaplan–Meier survival curves stratified by bedaquiline use are presented in Figure 7. Patients receiving bedaquiline demonstrated a higher probability of survival throughout the follow-up period compared with those who did not receive bedaquiline. The difference in survival between the two groups was statistically significant (log-rank test, *p* = 0.024). Cox proportional hazards analysis showed that bedaquiline use was associated with a reduced hazard of death (HR = 0.58, 95% CI: 0.35–0.94). These findings suggest that the inclusion of bedaquiline in treatment regimens was associated with improved survival among DR-TB patients in this cohort.

### 3.8. Adjusted and Crude Associations with Unfavorable Outcomes

Table 2 presents the crude and adjusted associations between demographic and clinical characteristics and unfavorable DR-TB treatment outcomes. In the unadjusted analysis, HIV-positive status, retreatment status, and treatment regimen were significantly associated with unfavorable outcomes. Patients who were HIV positive had higher odds of experiencing an unfavorable outcome compared with HIV-negative patients (OR 1.79; 95% CI: 1.33–2.30; *p* = 0.0001). Similarly, retreatment cases had increased odds of unfavorable outcomes compared with new cases (OR 1.80; 95% CI: 1.38–2.36; *p* < 0.0001). Patients receiving the individualized regimen also had higher crude odds of unfavorable outcomes compared with those on the six-month short regimen (OR 3.09; 95% CI: 2.02–4.74; *p* < 0.0001).

In the multivariable logistic regression model, after adjusting for potential confounders, HIV status remained independently associated with unfavorable treatment outcomes, with HIV-positive patients having higher odds of an unfavorable outcome compared with HIV-negative patients (aOR 1.36; 95% CI: 1.04–1.77; *p* = 0.025). Age was included as a continuous variable in the adjusted model and showed a modest but statistically significant association with unfavorable outcomes (aOR 1.01 per year increase; 95% CI: 1.00–1.02; *p* = 0.032). Other variables, including gender, resistance profile, treatment category, and treatment regimen, were not significantly associated with unfavorable outcomes after adjustment.

## 4. Discussion

This study highlights the epidemiological trends and treatment outcomes of drug-resistant tuberculosis (DR-TB) in Limpopo province between 2020 and 2024. RRTB predominated throughout the study period, comprising 76% of the cases and remaining stable throughout the years. This pattern is consistent with national surveillance data and studies from other high-burden settings in sub-Saharan Africa, where rifampicin-resistant tuberculosis remains the predominant form of drug resistance [16,17]. The proportions of MDRTB and pre-XDR-TB fluctuated within narrow ranges, while XDR-TB remained rare, suggesting a limited emergence of highly resistant strains but a persistent burden of RRTB in rural provinces [18,19,20].

The demographic profile of patients with DR-TB in Limpopo Province from 2020–2024 was dominated by males and individuals within economically productive age groups, consistent with the patterns reported in South Africa and other high-burden settings [8,10,21]. Nearly two-thirds of the cases occurred among men, likely reflecting a combination of occupational exposure, behavioral risk factors, and delayed health-seeking behavior. DR-TB was most common among adults 35 to 44 years of age, followed by those 25 to 34 years of age and 45 to 54 years of age. These highly mobile working age groups are more likely to experience sustained exposure and interruption of treatment, particularly in a cross-border province such as Limpopo, where migration for employment and trade is frequent. This mobility, combined with socioeconomic pressures, increases vulnerability to transmission and poor adherence, with important implications for continuity of care and long-term treatment outcomes. Although children represented a small proportion of cases, pediatric DR-TB remains epidemiologically significant and suggests ongoing household transmission and gaps in infection control. Pulmonary disease accounted for most of the cases, reflecting its higher transmissibility and easier detectability through routine diagnostic tools. In contrast, the low proportion of extrapulmonary disease may not necessarily indicate rarity but rather indicate underdiagnosis in rural settings. HIV co-infection was highly prevalent and independently associated with unfavorable treatment outcomes, underscoring the continued vulnerability of co-infected patients. This reflects both biological factors, such as immunosuppression and drug-drug interactions, and programmatic challenges, including delayed ART initiation and poor adherence. Together, these findings highlight the urgent need for strengthened TB/HIV integration and improved diagnostic capacity to address hidden burdens of extrapulmonary and pediatric DR-TB.

Rifampicin-resistant-TB accounted for more than three quarters of DR-TB cases throughout the five-year cohort. This pattern closely mirrors earlier reports from Limpopo and other South African provinces. In Limpopo, RR-TB comprised over 80% of resistance profiles [8,10]. The resistance profile remained largely stable, suggesting an established transmission of RR-TB strains in communities rather than transmission being driven solely by treatment failure or acquisition of resistance during therapy. The relative stability of resistance profiles over time reinforces previously documented challenges, particularly ongoing community-level transmission linked to delayed case findings, limited diagnostic access in rural districts, and high inter-district and cross-border population mobility [19,22]. Limpopo’s position as a cross-border province adjacent to Zimbabwe, Botswana, and Mozambique creates important challenges for tuberculosis control due to frequent population movement and treatment interruptions. Recent phylogenetic analyses from Limpopo have also identified transmission clusters of drug-resistant strains across districts, suggesting that both cross-border mobility and local transmission contribute to the persistence of DR-TB in the province [9].

### 4.1. Improvements in Treatment Outcomes, but Continued Gaps

Interestingly, treatment success improved steadily from 67% in 2020 to 78% in 2024, accompanied by a marked reduction in loss to follow-up. This improvement coincides with the widespread implementation of short, all-oral bedaquiline-containing regimens and reflects the strengthening of the performance of the provincial DR-TB program. A previous study in Limpopo province recorded an overall success rate of 59%, which increased to 65% after the introduction of all-oral bedaquiline based regimens [6]. Other similar patterns have been documented nationally, highlighting an overall 69% rate of successful treatment between 2015–2019 [23]. The sharp decline in loss to follow-up in Limpopo, from 13% in 2020 to 2% in 2024, reflects the impact of decentralized MDR-TB care and the transition to all-oral regimens. Previously, patients were managed in a single unit in Modimolle (Waterberg District), which served a population of more than 6 million and often required prolonged isolation and painful injectable therapy, leading to poor adherence and absconding. Following national policy, Limpopo established MDR-TB units in each of its five districts: Mankweng Hospital in Capricorn; Letaba Hospital in Mopani; Dr. C.N. Phatudi Hospital, serving Sekhukhune; Tshilidzini Hospital in Vhembe; and the Modimolle MDR-TB Unit in Waterberg, bringing care closer to communities, reducing travel and resource burdens, and allowing patients to remain within family and social support networks. Combined with the switch from six months of painful injections to bedaquiline-based all-oral regimens, these changes made treatment more acceptable and convenient, thus improving adherence and substantially reducing the loss to follow-up [24]. Despite these gains, overall treatment success remains suboptimal and falls below the WHO target of 90% success, underscoring the gap between current provincial outcomes and the global vision for 90% of 1990 for 2030, which calls for 90% of all TB patients to be diagnosed, 90% of those diagnosed to begin appropriate therapy and 90% of those treated to achieve successful outcomes [1].

The data from the 2020–2024 cohort results reflect continued improvements after the national adoption of short oral regimens, but overall mortality (15.6%), loss-to-follow-up (7.6%), and treatment failure (5.6%) remain a persistent challenge, particularly among older adults, HIV-positive individuals, and patients receiving individualized regimens. These risk patterns echo those previously reported in Limpopo and other South African regions, where co-infection compromises treatment response and increases vulnerability to poor clinical outcomes [8,23]. Prior evidence from the rural Limpopo bedaquiline outcomes study further supports our findings. In that study, BDQ-containing regimens significantly improved treatment success, accelerated sputum culture conversion, and reduced mortality compared to non-bedaquiline regimens. Similarly, shorter regimens with the BDQ base in this analysis were shown to be associated with treatment success [6]. Similar benefits have been reported in other South African settings and multinational cohorts, where the introduction of bedaquiline-based, all-oral regimens significantly improved treatment outcomes among patients with drug-resistant tuberculosis. In line with these observations, the present analysis also demonstrates that regimens incorporating bedaquiline were associated with higher treatment success and improved survival probabilities compared with regimens without bedaquiline. Together, these findings reinforce growing global evidence supporting the programmatic effectiveness of bedaquiline-containing regimens in improving DR-TB outcomes, particularly in high-burden settings [25,26].

Our results similarly show that individualized regimens, which are typically reserved for patients failing standard short regimens or those with advanced resistance patterns, were associated with poorer treatment outcomes. This observation has been reported in several studies from South Africa and other high-burden settings, where patients receiving individualized regimens often represent more clinically complex cases with extensive resistance, prior treatment exposure, and greater disease severity. Consequently, the poorer outcomes observed in these patients likely reflect the underlying complexity of their disease rather than the regimen itself. Similar findings have been documented in programmatic cohorts across sub-Saharan Africa and other high-burden regions, where individualized or longer regimens were associated with lower treatment success and higher mortality compared with standardized [27,28,29,30]. Together, these findings reaffirm the critical role of BDQ-based regimens and the need for provincial systems to ensure uninterrupted access to all-oral, short-course therapies. However, emerging evidence of bedaquiline and fluoroquinolone resistance poses a growing threat to maintaining these gains. Genomic surveillance data from Limpopo recently revealed concerns about levels of BDQ resistance (18%) and FLQ resistance (54%), with phylogenetic clustering indicating both localized and interdistrict transmission of resistant strains [9]. Broader literature shows that bedaquiline resistance in South Africa is generally reported in the range of 5–10%, depending on the cohort and province [31,32,33]. Similar resistance patterns have been observed in other multi-national studies [34,35]. The presence of transmission clusters previously reported in Limpopo underscores the reality that resistant strains are not only emerging due to inadequate treatment but are also actively transmitted in communities [9]. This creates a double burden: ongoing acquisition of resistance and propagation of already resistant strains. National data now show that bedaquiline resistance rates in South Africa are approaching or exceeding global estimates, highlighting the urgent need for routine phenotypic and genotypic drug susceptibility tests to guide regimen selection [32].

The relationship between previous drug history, patient category, and final treatment outcome demonstrates that prior treatment exposure primarily influences patient classification rather than directly determining treatment success. The strong association between the previous drug history and the patient category reflects the role of the treatment history in defining the complexity of the disease and retreatment status. However, the weak associations observed between previous drug history and treatment outcome, as well as between patient category and outcome, suggest that prior treatment alone is not a strong predictor of final outcomes in this cohort. This finding highlights the multifactorial nature of DR-TB treatment outcomes, where factors such as adherence, comorbidities, socioeconomic conditions, and access to healthcare can play a more decisive role than treatment history or patient classification alone.

Multivariable logistic regression analysis identified HIV infection as the only independent predictor of unfavorable outcomes in this cohort (aOR 1.36; 95% CI: 1.04–1.77; *p* = 0.025). In addition, increasing age showed a modest but statistically significant association with unfavorable outcomes when modelled as a continuous variable (aOR 1.01 per year increase; 95% CI: 1.00–1.02; *p* = 0.032). These findings underscore the important role of HIV in shaping treatment prognosis among DR-TB patients. This observation is consistent with national evidence indicating that the HIV/TB co-burden contributes to increased mortality and complicates treatment through immunosuppression, drug–drug interactions, and socioeconomic vulnerability. Other factors, including retreatment status and individualized treatment regimens, were associated with unfavorable outcomes in crude analyses but lost statistical significance after adjustment, suggesting that these variables may reflect underlying patient complexity rather than independently determining treatment outcomes [36].

These findings emphasize the need to strengthen TB/HIV integration, expand access to bedaquiline, and intensify early phase monitoring during the first three months of treatment to mitigate the high risk of mortality. Programmatic strategies must also address the cross-border continuity of care, given the geographic vulnerability of Limpopo, and address socioeconomic barriers to retention [10]. By sustaining improvements in treatment success and retention, while prioritizing HIV/TB services and regimen optimization, Limpopo can align provincial outcomes with national and global end-TB targets.

Kaplan–Meier survival analysis demonstrated that mortality occurred predominantly within the first 60 to 90 days of treatment, highlighting the early phase as the highest risk period. This pattern is consistent with previous DR-TB programmatic studies and reflects the high vulnerability of patients at the time of treatment, likely driven by advanced disease, delayed diagnosis, comorbid conditions, and early treatment-related toxicities [36]. Importantly, patients who survived the initial treatment period exhibited a substantially higher probability of remaining alive throughout the remainder of therapy, highlighting the critical importance of early clinical stabilization and close monitoring during the intensive phase of DR-TB treatment.

When survival was stratified by bedaquiline use, exposure showed a clear survival advantage for patients receiving bedaquiline-containing regimens. Patients consistently showed a higher probability of survival over time compared to those treated without bedaquiline. The early separation of the survival curves suggests that bedaquiline may confer a survival advantage soon after treatment initiation, a period known to carry the highest mortality risk in DR-TB. Although late follow-up in the non-bedaquiline group was characterized by few individuals at risk, the overall survival pattern supports growing evidence that bedaquiline-based regimens are associated with improved treatment outcomes and reduced mortality. These findings reinforce current WHO recommendations prioritizing bedaquiline-containing regimens for the treatment of drug-resistant tuberculosis and underscore the need to ensure early access to bedaquiline, particularly in high-burden and resource-limited settings.

### 4.2. Implications for Similar Regions

Limpopo province and other regions with comparable epidemiological profiles should prioritize interventions that strengthen early diagnosis (including expanded use of Xpert MTB/RIF, Xpert MTB/XDR, and WGS-supported surveillance) alongside robust integration of TB/HIV care, enhanced retention strategies, and access to individualized regimens informed by DST only when necessary. Genomic surveillance carried out in Limpopo has shown that local strengthening of diagnostic and analytical capacity is feasible and substantially improves the ability of rural settings to detect resistance, characterize transmission patterns and rapidly adapt treatment policies [9].

In general, this study contributes updated real-world evidence on DR-TB patterns in a predominantly rural, cross-border province with high HIV burden, adding contemporary relevance to global discussions on DR-TB control. By integrating epidemiological trends with analyses of treatment outcome patterns and emerging insights into drug resistance dynamics, the findings provide a comprehensive overview of DR-TB in Limpopo province. Importantly, these patterns extend beyond the local context and are highly relevant not only for Limpopo but also for other regions characterized by rurality, mobility, limited health care resources, and a high burden of co-infection with TB/HIV. Sustaining and expanding recent gains in DR-TB control will require continued investment in diagnostic modernization, responsible stewardship of new and existing anti-TB drugs, and strengthening the surveillance-enabling infrastructure to support timely detection, effective treatment, and interruption of transmission in Limpopo and comparable settings.

### 4.3. Strength of the Study

The study used a large provincial data set that included all laboratory confirmed DR-TB cases in Limpopo province over a five-year period, which increased representativeness and allowed robust analysis of trends and treatment results in the rural high-burden setting.

The use of routinely collected data from primary centers up to tertiary hospital in Limpopo provided real world insight into health system performance, increasing the policy relevance of the findings.

In addition, the study period managed to capture changes after the scaling-up of short all-oral regimens, and applied multivariate analysis, which allows identification of independent predictors and unfavorable outcomes.

### 4.4. Limitations

This study has several limitations that should be considered when interpreting the findings. First, the retrospective design relied on routinely collected programmatic data from the EDRWeb data, which are subject to incompleteness, reporting delays, and potential data entry errors [10]. Although the EDRWeb database represents the main provincial source for DR-TB surveillance, missing or inconsistently recorded variables, particularly on treatment outcomes, HIV clinical parameters (such as ART status, CD4 count, or viral load), and detailed previous treatment history may have introduced bias or limited the precision of some analyses. The study also excluded patients with missing outcome data from the outcome analysis, which may also result in selection bias if these patients differed systemically from those with complete records.

Secondly, the study did not capture social, economic, or behavioral factors such as treatment adherence, alcohol use, or socioeconomic hardship that are known to influence DR-TB outcomes. The inability to adjust for these confounders may limit the interpretation of some associations observed between demographic or clinical variables and treatment outcomes.

Third, resistance categories were based on routine programmatic definitions, and the absence of complete phenotypic and genotypic drug susceptibility data may have resulted in some misclassification, particularly for pre-XDR and XDR-TB, contributing to variability in outcomes.

Fourth, a small proportion of records lacked final treatment outcome information due to patient transfer or ongoing treatment at the time of database extraction. Although these cases were excluded from outcome regression analyses, they were retained as right-censored observations in the survival analysis to minimize bias.

Finally, the study reflects only diagnosed and reported cases of DR-TB. Limited access to diagnostic services, particularly in rural areas, and disruptions in the use of health services, especially during the hard lockdown periods of COVID-19 (2020–2021), may have resulted in underdiagnosis and under notification of DR-TB during the study period. Thus, the true burden of DR-TB in the province may be higher than reported.

Despite these limitations, the study provides up-to-date, valuable evidence on DR-TB trends and treatment outcomes in a rural, high mobility, resource-constrained province, offering insights that are relevant for similar regions worldwide.

## 5. Conclusions

This study shows that DR-TB in Limpopo remains dominated by forms of RR-TB and MDR-TB, with gradual improvements in treatment outcomes following the roll-out of short all-oral regimens. However, persistent mortality, loss to follow-up, and increased vulnerability among HIV-positive and older patients highlight ongoing programmatic gaps. Survival analyses further indicate that patients receiving bedaquiline-containing regimens experience improved survival probabilities compared with those treated without bedaquiline, reinforcing the clinical value of these regimens. From a health policy perspective, strengthening access to bedaquiline-based treatment, improving TB/HIV service integration, expanding diagnostic and drug susceptibility testing capacity, and enhancing treatment retention strategies are essential to improve outcomes in Limpopo. These insights are relevant not only for Limpopo but also for other rural and high-mobility settings in South Africa facing similar DR-TB control challenges.

## Figures and Tables

**Table 1:** Demographic and clinical characteristics of patients enrolled in DR-TB treatment in Limpopo from 2020–2024 (*n* = 1240).

Variables		RR-TB *n* (%)	MDR-TB *n* (%)	Pre-XDR-TB *n* (%)	XDR-TB *n* (%)	*p*-Value	Total *n*
**Gender**	Male	584 (76.1)	135 (17.6)	41 (5.3)	7 (1.0)	0.508	767
	Female	367 (77.6)	77 (16.3)	21 (4.4)	8 (1.7)		473
**Age group**	0–14	19 (76.0)	4 (16.0)	2 (8.0)	0 (0)	0.434	25
	15–24	94 (78.3)	20 (16.7)	4 (3.3)	2 (1.7)		120
	25–34	224 (79.2)	44 (15.5)	11 (3.9)	4 (1.4)		283
	35–44	303 (75.9)	74 (18.5)	16 (4.0)	6 (1.5)		399
	45–54	190 (72.0)	51 (19.3)	21 (8.0)	2 (0.8)		264
	55–64	84 (79.2)	17 (16.0)	4 (3.8)	1 (0.9)		106
	≥65	37 (86.0)	2 (4.7)	4 (9.3)	0 (0)		43
**Pulmonary/Extrapulmonary**	Pulmonary	892 (76.4)	204 (17.5)	58 (5.0)	13 (1.1)	0.535	1167
	Extrapulmonary	44 (81.5)	6 (11.1)	2 (3.7)	2 (3.7)		54
	Both	4 (80)	0 (0)	1 (20)	0 (0)		5
	Unknown	11 (78.6)	2 (14.3)	1 (7.1)	0 (0)		14
**Year**	2020	196 (75.4)	45 (17.3)	14 (5.4)	5 (1.9)	0.920	260
	2021	162 (76.1)	41 (19.2)	7 (3.3)	3 (1.4)		213
	2022	203 (78.8)	38 (14.7)	15 (5.8)	2 (0.8)		258
	2023	216 (76.6)	48 (17.0)	16 (5.7)	2 (0.7)		282
	2024	174 (76.7)	40 (17.6)	10 (4.4)	3 (1.3)		227
**Patient category**	New	635 (79.4)	131 (16.4)	29 (3.6)	8 (1.0)	**<0.001**	803
	Relapse	111 (73.0)	36 (23.7)	5 (3.3)	0 (0.0)		152
	Treatment after failure	98 (66.2)	24 (16.2)	22 (14.9)	4 (2.7)		148
	Treatment after LTFU	72 (77.4)	14 (15.1)	4 (4.3)	3 (3.2)		93
	Other	35 (79.5)	7 (15.9)	2 (4.6)	0 (0)		44
**HIV status**	Positive	588 (76.6)	130 (16.9)	42 (5.5)	8 (1.0)	0.394	768
	Negative	337 (75.9)	81 (18.2)	19 (4.3)	7 (1.6)		444
	Unknown	26 (92.8)	1 (3.6)	1 (3.6)	0 (0)		28
**Previous drug history**	New	625 (79.0)	129 (16.3)	29 (3.7)	8 (1.0)	**0.026**	791
	Previously exposed	298 (71.6)	79 (19.0)	32 (7.7)	7 (1.7)		416
	Unknown	28 (84.8)	4 (12.1)	1 (3.0)	0 (0)		33
**Regimen**	Short regimen (6-months)	214 (77.4)	48 (17.4)	14 (5.1)	0 (0)	**<0.001**	276
	Short regimen (9–11 months)	607 (82.0)	124 (16.8)	6 (0.8)	3 (0.4)		740
	Individualized regimen	130 (58.0)	40 (17.8)	42 (18.8)	12 (5.4)		224

**Table 2:** Distribution and logistic regression analysis of variables associated with DR-TB treatment outcomes (*n* = 1151).

Variable	Favorable Outcomes *n* (%)	Unfavorable Outcomes *n* (%)	Crude OR (95% CI)	Crude OR *p*-Value	Adjusted OR (95% CI)	Adjusted OR *p*-Value
**Gender**						
**Female (Ref)**	301 (68.0)	142 (32.0)	Ref		Ref	
**Male**	507 (71.6)	201 (28.4)	0.84 (0.65–1.08)	0.187	0.98 (0.72–1.18)	0.537
HIV status *						
**Negative (Ref)**	315 (77.0)	95 (23.0)	Ref		Ref	
**Positive**	479 (67.0)	241 (33.0)	1.79 (1.33–2.30)	0.0001	1.36 (1.04–1.77)	0.025
**Resistance profile**						
**RR-TB (Ref)**	635 (71.6)	251 (28.4)	Ref		Ref	
**MDR-TB**	127 (65.5)	67 (34.5)	1.30 (0.93–1.80)	0.143	0.95 (0.68–1.33)	0.767
**Pre-XDR-TB**	38 (66.7)	19 (33.3)	1.29 (0.73–2.27)	0.448	0.92 (0.51–1.67)	0.787
**XDR-TB**	7 (58.3)	5 (41.6)	3.21 (0.86–12.06)	0.128	0.59 (0.12–2.82)	0.509
**Treatment category**						
**New (Ref)**	559 (74.7)	190 (25.3)	Ref		Ref	
**Retreatment**	222 (61.7)	138 (38.3)	1.80 (1.38–2.36)	<0.0001	1.07 (0.82–1.40)	0.612
**Treatment regimen**						
**Short regimen (6 months) (Ref)**	174 (79.9)	44 (20.1)	Ref		Ref	
**Short regimen (9–11 months)**	512 (71.2)	207 (28.7)	1.65 (1.14–2.38)	0.007	1.22 (0.88–1.69)	0.232
**Individuali z ed regimen**	122 (57.0)	92 (43.0)	3.09 (2.02–4.74)	<0.0001	1.22 (0.87–1.71)	0.250

* Patients with unspecified HIV status were excluded from the multivariable regression analysis to allow HIV status to be treated as a binary variable.

## Data Availability

The original contributions presented in this study are included in the article/Supplementary Material. Further inquiries can be directed to the corresponding authors.

## Supplementary Materials

The following supporting information can be downloaded at: https://www.mdpi.com/article/10.3390/tropicalmed11040100/s1.

## Abbreviations

The following abbreviations are used in this manuscript:

TB	Tuberculosis
RR-TB	Rifampicin resistant tuberculosis
MDR-TB	Multidrug resistant tuberculosis
Pre-XDR-TB	Pre-extensively drug resistant tuberculosis
XDR-TB	Extensively drug resistant tuberculosis
BDQ	Bedaquline
DR-TB	Drug resistant tuberculosis
HIV	Human immunodeficiency virus
EDRWeb	Electronic Drug-Resistant Tuberculosis Register Web
